# Functional characterization of a cell wall invertase inhibitor StInvInh1 revealed its involvement in potato microtuber size *in vitro*

**DOI:** 10.3389/fpls.2022.1015815

**Published:** 2022-10-03

**Authors:** Cheng Liu, Shuting Hu, Shuyi Liu, Weiling Shi, Debin Xie, Qi Chen, Hui Sun, Linjing Song, Ziyu Li, Rui Jiang, Dianqiu Lv, Jichun Wang, Xun Liu

**Affiliations:** Integrative Science Center of Germplasm Creation in Western China (CHONGQING) Science City, Chongqing Key Laboratory of Biology and Genetic Breeding for Tuber and Root Crops, Engineering Research Center of South Upland Agriculture, Ministry of Education, Southwest University, Chongqing, China

**Keywords:** potato, cell wall invertase, invertase inhibitor, sucrose metabolism, microtuber

## Abstract

Cell wall invertase (CWI) is as an essential coordinator in carbohydrate partitioning and sink strength determination, thereby playing key roles in plant development. Emerging evidence revealed that the subtle regulation of CWI activity considerably depends on the post-translational mechanism by their inhibitors (INHs). In our previous research, two putative INHs (StInvInh1 and StInvInh3) were expected as targets of CWI in potato (*Solanum tubersum*), a model species of tuberous plants. Here, transcript analysis revealed that *StInvInh1* showed an overall higher expression than *StInhInh3* in all tested organs. Then, *StInvInh1* was further selected to study. In accordance with this, the activity of *StInvInh1* promoter increased with the development of leaves in plantlets but decreased with the development of microtubers *in vitro* and mainly appeared in vascular bundle. The recombinant protein StInvInh1 displayed inhibitory activities on the extracted CWI *in vitro* and StInvInh1 interacted with a CWI StcwINV2 *in vivo* by bimolecular fluorescence complementation. Furthermore, silencing *StInvInh1* in potato dramatically increased the CWI activity without changing activities of vacuolar and cytoplasmic invertase, indicating that StInvInh1 functions as a typical INH of CWI. Releasing CWI activity in *StInvInh1* RNA interference transgenic potato led to improvements in potato microtuber size in coordination with higher accumulations of dry matter *in vitro.* Taken together, these findings demonstrate that *StInvInh1* encodes an INH of CWI and regulates the microtuber development process through fine-tuning apoplastic sucrose metabolism, which may provide new insights into tuber development.

## Introduction

Invertases are key metabolic enzymes regulating sink activity through the hydrolytic cleavage of sucrose into glucose and fructose, which are used for diverse metabolic and signaling pathways to regulate plant growth and development (Ruan, 2014). Invertases are classified as cell wall/apoplastic invertase (CWI), vacuolar invertase (VI) and cytosolic neutral/alkaline invertase (CI) on the basis of their cellular targets (Sturm, 1999). The conserved domain of CWI and VI protein belong to glycoside hydrolase family 32 (GH32) enzymes with an optimal pH of 3.5–5.0. Both CWIs and VIs are glycosylated enzymes and intrinsically stable. However, CIs are not glycosylated and clustered to GH100 with an optimal pH of 6.8–9.0 (Coculo and Lionetti, 2022). CIs function in oxidative stress defense (Xiang et al., 2011), and cellulose biosynthesis (Rende et al., 2017; Barnes and Anderson, 2018). VIs often play major roles in hexoses accumulation and osmotic regulation (Klann et al., 1996; Bhaskar et al., 2010; Wang et al., 2010). Molecular genetic studies showed that CWIs are required for seed development and fruit set in some instances, probably by controlling cell division in endosperm and embryo. An endosperm-specific *CWI* mutation in maize resulted in a miniature seed phenotype owing to reduced mitotic activity and cell size in the endosperm (Miller and Chourey, 1992; Cheng et al., 1996; Vilhar et al., 2002). A similar phenotype of *CWI* mutation in seed development was documented in rice (Wang et al., 2008) and tomato (Zanor et al., 2009). Conversely, constitutive expression of *CWI* genes dramatically increases grain yield and total starch content in maize (Li et al., 2013). However, ectopic expression of a CWI gene *GIF1* with the CaMV35S or rice *waxy* promoter resulted in smaller grains in rice, whereas overexpression of *GIF1* driven by its native promoter increased grain production (Wang et al., 2008). These results indicate that CWIs function as determinates of crop yield or production in a gene-dosage-dependent manner or spatial-temporal dependent manner in different plants.

Earlier research on the control of CWI/VI activities mainly focused on transcriptional regulation by modulating their transcripts. However, the proteins of CWI/VIs are intrinsically stable due to glycosylation (Ruan et al., 2010). Thus, their activities are also regulated largely at the protein level. Recent studies have shown that the CWI/VI activities were regulated on the post-translational mechanism through protein–protein interaction between CWIs/VIs and their inhibitors (INHs). The INHs directly target the active site of invertase and compete with sucrose (the substrate of the invertase) for the same binding site (Hothorn et al., 2010). This protein was discovered as early as 1961 when studying the dynamics of potato tuber invertases (Schwimmer et al., 1961). While, the first plant INH of CWI was isolated from tobacco (Greiner et al., 1998). Subsequently, the physiological functions of these INHs in the regulation of seed development have been well documented in maize (Bate et al., 2004; Chen et al., 2019), tomato (Jin et al., 2009), *Arabidopsis* (Su et al., 2016), and soybean (Tang et al., 2017), reflecting that it is a promising strategy to improve seed yield *via* fine-tuning manipulation of CWI activities.

Potato, a model species of tuberous plants, is the most important non-cereal staple crop that widely used throughout the world. The improvement of potato yield potential remains a major challenge for modern agriculture. To understand the effect of the elevating CWI activity on potato tuber development, overexpression of apoplastic yeast invertase in potato was performed. Initially, the constitutive overexpression of apoplastic yeast invertase caused the plants to appear to be under stress and yield penalty (Heineke et al., 1992; Büssis et al., 1997). Subsequently, the tuber-specific overexpression of apoplastic yeast invertase resulted in an increase in tuber size and total yield owing to increased water content (Sonnewald et al., 1997; Hajirezaei et al., 2000; Ferreira and Sonnewald, 2012), further indicated that the CWI activity need to be finely regulated to improve tuber development. Although increasing CWI activities *via* these approaches have been studied for the formation and development of tubers, the CWI activities have been finely regulated at the molecular level, especially during the stolon-tuber formation period, and its influence on the formation and development of tubers still remains unknown.

In our previous study, four putative *INHs* were isolated from potato (Liu et al., 2010). Among them, both StInvInh2A and StInvInh2B function as INHs of VI to diminish cold-induced sweetening in cold-stored tubers (Liu et al., 2010; Liu et al., 2013; Lin et al., 2015). However, the physiological functions of another two putative INHs of CWI are still unknown. In this study, StInvInh1 was identified as an important INH of CWI related to biological processes during potato growth and development. In order to have a better understanding of the importance of the apoplastic sucrose metabolism for potato development, the transgenic plants with released CWI activities were made by silencing *StInvInh1*. Knockdown of *StInvInh1* in potato exclusively increased the CWI activity and led to improvement in potato microtuber size and weight in coordination with higher accumulations of dry matter *in vitro*. The results will help clarify the function of endogenous CWI activity in regulating tuber development and provide a potential avenue for improving tuber production.

## Materials and methods

### Plant materials and growth conditions

The wild-type (WT) and transgenic plantlets were multiplied in tissue culture on semisolid (7 g L^−1^ agar) Murashige & Skoog (MS) medium with 4% sucrose and incubated at 20 ± 1°C with a photoperiod of 16/8 h day/night (light intensity 100 μmol m^−2^ s^−1^). The second or the third single stem nodes from the uppermost of 4-week-old plantlets were transferred to the microtuber induction MS medium with 8% sucrose, 0.7% agar, and 0.2% activated carbon and then incubated at 20 ± 1°C with 8/16 h day/night photoperiods (Liu and Xie, 2001). The characteristics of microtuberization *in vitro* were investigated by using 200 plantlets for each line. Four-week-old microtubers were harvested for microtuber size observation. The samples of various organs in potato were prepared for the expression patterns of *StInvInh1* and *StInvInh3* in our previous study (Liu et al., 2011).

### Isolation and analysis of the *StInvInh1* promoter sequence

A pair of specific primers were designed to amplify 5’-flanking sequences of *StInvInh1* based on the *StInvInh1* (Soltu.DM.12G001750.1) genome sequence. The CTAB method was used to isolate the genomic DNA from leaves of three-week-old plantlets of potato cultivar E3. The final PCR products were gel-purified and cloned into the pEASY simple blunt vector (BioGene, Beijing, China), and subjected to sequencing. Putative cis-elements in the *StInvInh1* promoter sequence were searched using the Plant-CARE (http://bioinformatics.psb.ugent.be/webtools/plantcare/html) and PLACE (http://www.dna.affrc.go.jp/PLACE/signalscan.html) databases.

### Transient transcription dual-LUC assay

For the promoter activity assay, the *StInvInh1* promoter sequence was subcloned into the pGreenII 0800-LUC double-reporter vector. Dual-LUC assays were performed on *N. benthamiana* plants as described previously (Liu et al., 2021). The Firefly luciferase (LUC) and Renillia (REN) luciferase activity of the plant protein extract was analyzed by a Promega GloMax 20/20 Luminometer (Promega, Madison, USA) using the dual luciferase assay kit (Vazyme, Nanjing, China). The results were calculated by the LUC/REN ratio. At least three measurements were calculated for each assay, and three individual replicates were performed.

### Vector construction and plant transformation

For constructing the *StInvInh1* promoter:: *GUS* binary vector, an approximate 2.2-kb fragment from -2157 to the translation start codon was sub-cloned into the pBI121 vector (Invitrogen, Carlsbad, CA, USA). For constructing the *CaMV35S*:: *StInvInh1* RNAi vector, a 348-bp fragment starting from 69 bp downstream of the start codon of the *StInvInh1* cDNA was subcloned into pENTR/D cloning vector (Invitrogen, USA). The fragment was further subcloned into pHellsGate8 vector with the recombination method (Helliwell et al., 2002). Sequences in the recombinant pHellsGate8-*StInvInh1* plasmid were confirmed by restriction digestion (*Xho* I and *Xba* I) and sequencing of inserts to ensure that the *StInvInh1* sequences recombined in sense and antisense orientations. The resulting constructs were transformed into the *Agrobacterium tumefaciens* GV3101 strain and transformed into potato E3 as previously described (Liu et al., 2013). The four-week-old plantlets and microtubers *in vitro* were sampled, used immediately for GUS staining, or frozen in liquid nitrogen and stored -80°C for GUS expression analysis. The characteristics of microtuberization *in vitro* were investigated by using 200 plantlets for each RNAi line. Four-week-old microtubers were harvested for observation of the size of microtubers.

### Histochemical determination of GUS activity

Fresh samples (the plantlets or microtubers slices) were subjected to the X-Gluc solution (Sangon, Shanghai, China) for histochemical determination of GUS activity (Liu et al., 2017).

### RNA extraction and RT-qPCR

All the samples are quick-frozen in liquid nitrogen and stored in -80°C refrigerator. The tissues were grounded, and total RNA was extracted using the RNA purification kit (Tiangen, Beijing, China). The quantitative RT-PCR (RT-qPCR) was performed as previously described by Liu et al. (2010). The procedure was as follows: 95°C 30 s, 40 cycles, 95°C 15 s, 55°C 30 s, 72°C 5 s. The specificity of the individual PCR amplification was confirmed by a dissociation curve protocol from 60 to 95°C and electrophoresis on agarose gel after the last cycle of real-time qPCR. Potato gene *ef1α* (AB061263) was used as an internal control (Nicot et al., 2005). All primers used in this study are presented in **Supplemental Table S1**.

### Determination of invertase activity *in planta* and *in vitro*

Samples of plantlets and microtuber slices were fixed with the fixation buffer (2% paraformaldehyde, 2% polyvinylpyrrolidone 40, 10 mM dithiothreitol, pH=7.0) for 1 h at 4°C. After fixation, samples were washed overnight in water and refreshed at least five times to get rid of the soluble sugar. The analyses of invertase activity *in planta* and *in vitro* were performed as previously described (Sergeeva et al., 2006; Liu et al., 2013). Samples of plantlets and microtuber slices were ground in liquid nitrogen.

### Quantification of the fresh and dry weight, the contents of dry matter, starch, sucrose, fructose and glucose in microtubers

One hundred four-week-old microtubers were collected and weighed as one biological replicate, and three biological replicates were sampled for each transgenic line. The fresh weight was determined by the average weight of the microtubers. Then, the microtubers were dried at 80°C for 48 h in an oven. The dry weight was determined by calculating the average weight of the dried microtubers. Dried tuber samples were grounded to fine powder. The starch, sucrose, fructose and glucose in each sample were extracted and determined following the instructions provided with the starch, sucrose, fructose and glucose assay kits (Solarbio, Beijing, China), respectively.

### Functional assays of recombinant *StInvInh1*

The coding sequence (without signal peptide) of *StInvInh1* was sub-cloned into expression vector E6 (GenScript, USA) with an N-terminal 6× His tag. The *E. coli* strain Rosetta-gami™ (DE3) (Novagen, USA) was used as host for the protein expression. Expression and purification of recombinant StInvInh1 protein was performed following the protocol reported by Liu et al. (2010). Assay for inhibitor function of recombinant StInvInh1 protein *in vitro* were performed as described by Link et al. (2004).

### Protein-protein interaction between *StInvInh1* and *StcwINV2*

For the bimolecular fluorescence complementation (BiFC) analysis, the full-length cDNA fragments of *StInvInh1* and *StcwINV2* without their stop codon were amplified and subcloned into the BiFC vectors, respectively (Walter et al., 2004). The subsequent constructs were transformed into the BY-2 cells by particle bombardment as previously described (Liu et al., 2010). Afterwards, the transformants were incubated at 26°C for 24 h in dark. Fluorescence signals for YFP (excitation 514 nm) of the successful transformants were detected and recorded by confocal laser scanning microscope (LSM510 Meta, Zeiss, Germany).

### Statistical analyses

One-way ANOVA test was accomplished for data analyses using IBM SPSS Statistics 20. The student’s t-test was carried out using the software in Excel 2017 (Microsoft, USA). Data are means ± SD from at least three independent replicates. Differences depicted as “*” and “**” were accepted as significant at P < 0.05 or 0.01.

## Results

### Expression patterns of *StInvInh1* in various organs of potato

**Figure 1:**
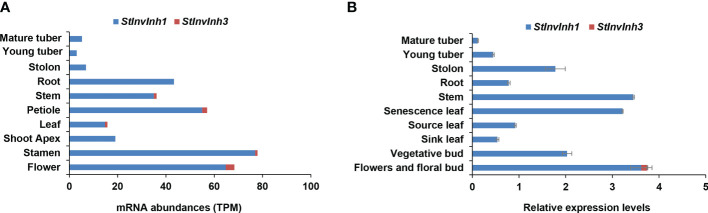
Relative expression levels of putative cell wall invertase inhibitor genes in various organs of potato plants. **(A)** The mRNA abundances of *StInvInh1* and *StInvInh3* are estimated from RNA-seq data of potato genotype RH *in silico*. **(B)** The relative expression levels of *StInvInh1* and *StInvInh3* genes are presented in relation to the expression levels of *ef1α* (AB061263) transcripts (100) by RT-qPCR. Data are means ± SD of three independent samples.

In our previous study, two putative cell wall invertase inhibitor genes (*StInvInh1* and *StInvInh3*) were isolated (Liu et al., 2010). To compare the expression levels of *StInvInh1* and *StInvInh3* in various organs, their transcripts were estimated by RNA-seq data from potato genotype RH (http://spuddb.uga.edu/). The results revealed a low or undetectable expression of *StInvInh3* in various organs, whereas *StInvInh1* showed an overall higher expression, with the highest transcript levels in flower and stamen (**Figure 1A**). The expression patterns of *StInvInh1* and *StInvInh3* were further analyzed in various organs by RT-qPCR (**Figure 1B**). Consistent with the RNA-seq data, RT-qPCR analysis showed that *StInvInh3* was only detectable in flower and flower bud with a low abundance, whereas *StInvInh1* exhibited constitutive expression with a higher expression level in flower and flower buds, senescence leaves, and stems. Notably, the mRNA level of *StInvInh1* decreased with the development of tubers from stolon to tuber. These results indicated that *StInvInh1* may work as an important *INH* gene involved in biological processes of potato growth and development. Thus, *StInvInh1* was selected for further study.

**Figure 2:**
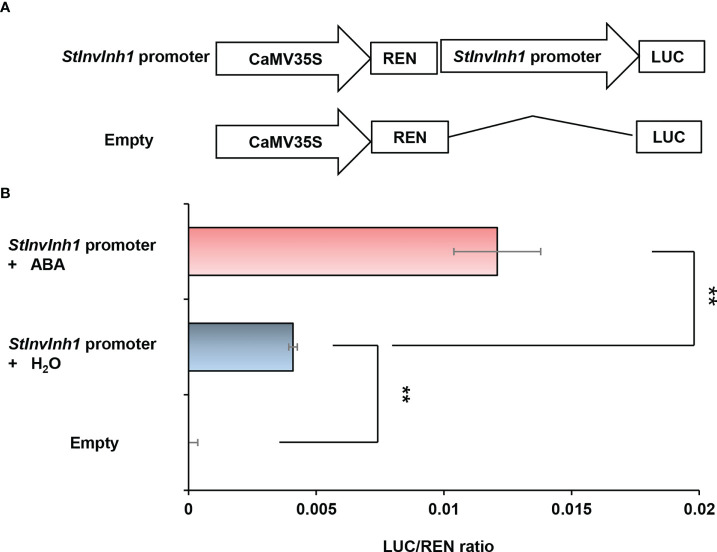
Estimation of the promoter activity of the 2.2-kb *StInvInh1* promoter sequence by the dual-luciferase reporter assays. **(A)** Schematic representation of the double-reporter plasmids used in the assay. The double-reporter plasmids contain the *StInvInh1* or empty promoter fused to LUC luciferase and REN luciferase driven by CaMV35S. **(B)** The promoter activity of the 2.2-kb *StInvInh1* promoter sequence. The dual-luciferase reporter vectors were introduced into tobacco leaves by *Agrobacterium tumefaciens* strain GV3101. The infiltrated tobacco leaves were spayed by ABA (50 mM) or H_2_O. After 48 h from the infiltration, LUC and REN luciferase activities were assayed. Each value represents the means of three biological replicates, and vertical bars represent the S.D. ^**^Significant differences in values (P < 0.01) by Student’s t-test.

For more detail on *StInvInh1* expression, its promoter activity was further analyzed. According to the *StInvInh1* gene sequence (Soltu.DM.12G001750.1) in potato reference genome of *S. tuberosum* group *Phureja* clone DM 1-3 (Pham et al, 2020), an approximate 2.2 kb length of 5’-flanking sequences of *StInvInh1* was isolated from E3 genomic DNA (**Figure S1**). The sequencing results indicated that an abscisic acid (ABA)-responsive element (ABRE), a methyl jasmonate-responsive element (CGTAC-motif), an auxin-responsive element (TGA-element), a gibberellin-responsive element (P-box), a stress-responsive element (TC-rich element), and several light-responsive elements (I-box and Box4) were predicted over the 2.2-kb promoter region, suggesting that the expression of *StInvInh1* may be regulated by different physiological and environmental factors. Subsequently, to estimate the promoter activity of the isolated promoter sequence, the transient expression assays were performed using the dual-luciferase reporter assays. The dual luciferase reporter plasmids harboring the 2.2-kb *StInvInh1* promoter sequence were fused to *LUC*, and the *REN* driven by the CaMV35S promoter was used as an internal control (**Figure 2A**). Compared with the empty control, the promoter activity of the 5’-flanking sequence of *StInvInh1* was detectable. In addition, its promoter activity was activated by ABA (**Figure 2B**). These results suggested that the 2.2-kb promoter region of *StInvInh1* is a functional promoter.

**Figure 3:**
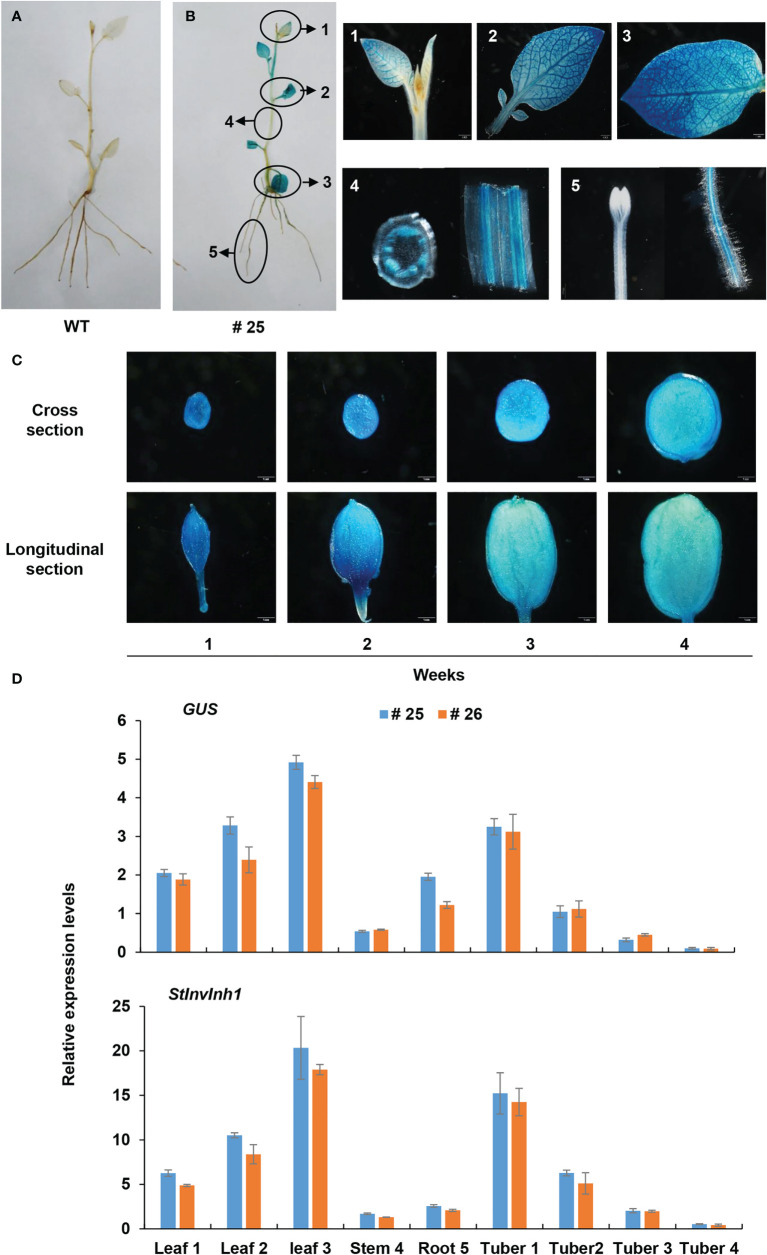
Expression pattern of GUS under the control of the *StInvInh1* promoter. **(A)** GUS staining in plantlets of WT; **(B)** GUS staining in plantlets of a representative transgenic line (#25); **(C)** GUS staining in micro-tubers of a representative transgenic line (#25); **(D)** The relative expression levels of *GUS* and *StInvInh1* in two representative transgenic line (#25 and #26). The 4-week-old plantlets and micro-tubers *in vitro* were subjected to the GUS staining and *GUS* expression. Leaves, stems, roots and developing micro-tubers were observed. Each repeat sample contains at least 6 plantlets or micro-tubers. Each sample was distributed into two groups. One was used for histochemical GUS staining; the other was frozen in liquid nitrogen and stored at -80°C for *GUS* expression analysis. The expression level of potato *ef1α* (AB061236) was set as 100 and used for normalization. Each data point is mean value of triplicate readings.

Then, the promoter sequence was fused to the coding sequence of β-glucuronidase (GUS) to construct a vector denoted as *pInh1::GUS*, and transformed to potato E3. The four-week-old plantlets of the transgenic lines were stained blue by X-Gluc solution (**Figure 3**), further demonstrating that the 5’-flanking sequence of *StInvInh1* possessed promoter activity. The GUS signal was detected in almost all tested organs and seems to mainly appear in the vascular bundles of stems. The strength of the GUS signal increased with the development of leaves in plantlets but decreased with the development of micro-tubers *in vitro* (**Figures 3B, C**). The RT-qPCR analysis revealed that the mRNA abundances of *GUS* were in accordance with the histochemical assay. Furthermore, a similar expression pattern was also observed between *GUS* and *StInvInh1* in transgenic lines (**Figure 3D**), indicating that the promoter function of the isolated sequence is similar with that of the *StInvInh1* native promoter.

### Inhibitory functions of StInvInh1

**Figure 4:**
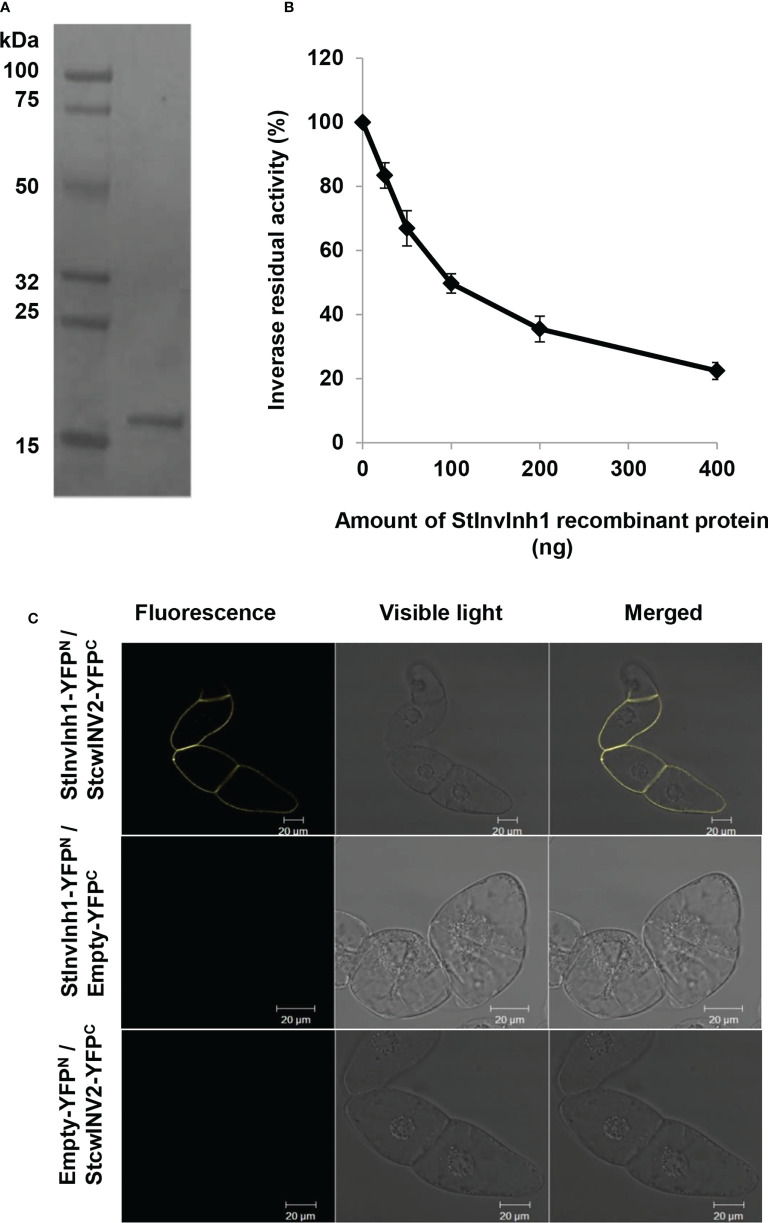
Inhibitory functions of StInvInh1. **(A)** The purified recombinant StInvInh1 protein; **(B)** Inhibitory effects of recombinant StInvInh1 protein on CWI activity in potato. Dose-dependent effects of StInvInh1 protein on CWI activity isolated from potato leaves are shown. Residual invertase activity was measured at pH 4.6 and 37°C after 30 min pre-incubation of the recombinant StInvInh1 protein and crude CWI in potato leaves. **(C)** Interaction of StInvInh1 and StcwINV2 proteins in tobacco BY-2 cells by bimolecular fluorescence complementation. Tobacco BY-2 cells were transformed by particle bombardment with a set of constructs for StInvInh1-YFP^N^ and StcwINV2-YFP^C^, StInvInh1-YFP^N^ and empty-YFP^C^, empty -YFP^N^ and StcwINV2-YFP^C^, respectively.

To determine whether StInvInh1 is a functional INH of CWI, the recombinant StInvInh1 protein’s inhibitory activity was tested by incubating with CWI fractions from potato leaves. Heterologous expression in the *E. coli* strain Rosetta-gami™ (DE3) yielded N-terminal His fusion proteins of StInvInh1. The purified StInvInh1 was recovered by Ni-TED affinity chromatography (**Figure 4A**). A decrease in the CWI activity levels was observed upon increasing the recombinant StInvInh1 protein concentration (**Figure 4B**), suggesting the StInvInh1 function as an INH of CWI *in vitro*.

A further confirmation of the protein–protein interaction between StInvInh1 and CWI was performed in living plant cells using the BiFC. Since *StcwINV2* was potentially co-expressed with *StInvInh1* in tested organs (Liu et al., 2011), it was selected as a representative of CWIs in potato. Sets of pSPYNE-35S and pSPYCE-35S constructs of *StInvInh1* and *StcwINV2* were transformed the tobacco BY-2 cells. A fluorescence signal was observed when *StcwINV2-YFP^C^* was co-expressed with *StInvInh1-YFP^N^*, while the control cells transformed with any combination with empty vectors produced no fluorescence (**Figure 4C**). The results indicated that StInvInh1 interacted with StcwINV2 *in vivo*. Taken together, these results clearly defined the StInvInh1 targeted CWI *in situ.*

### Silencing *StInvInh1* expression specifically releases CWI activities in transgenic plantlets

**Figure 5:**
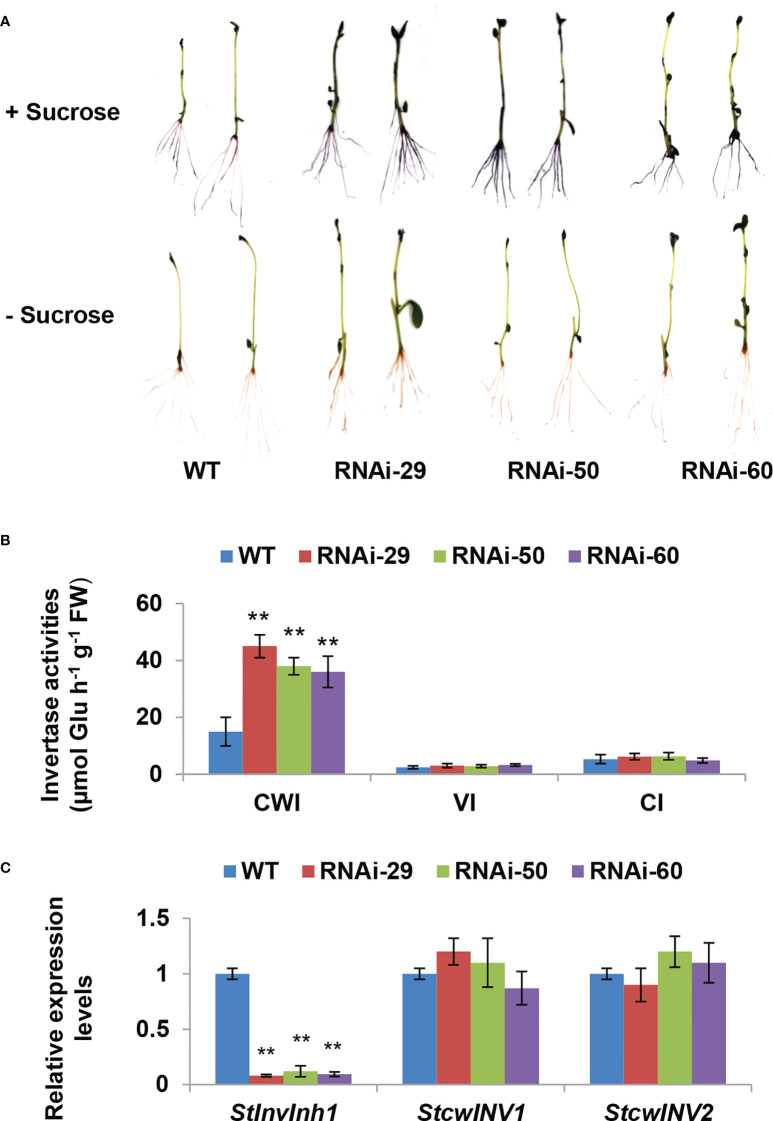
Silencing *StInvInh1* expression specifically releases CWI activity in transgenic plantlets. **(A)** Histochemical staining of NBT indicating the increased acid invertase activities in the RNAi plantlets. **(B)** The invertase activity determined by enzyme assay *in vitro* indicating the significantly increased the CWI activity in the RNAi plantlets without impacting the activities of VI and NI. **(C)** RT-qPCR analysis revealed that *StInvInh1* was suppressed in the RNAi plantlets without impact on mRNA levels of the two CWI genes, *StcwINV1* and *StcwINV2*. The relative expression levels of *StInvInh1*, *StcwINV1* and *StcwINV2* are presented in relation to the expression level of *ef1α* (AB061263) transcripts (100). The relative expression level of each gene and each enzyme activity in transgenic lines was compared with that in wild-type control E3. Each value was the mean ± SD of three biological replicates. **Significant differences in values (P < 0.01) by Student’s t-test.

To investigate the physiological roles of *StInvInh1 in vivo*, transgenic potatoes were generated by a RNA interference (RNAi) approach to downregulate the *StInvInh1* mRNA abundances. No obvious phenotypic difference was observed in four-week-old plantlets between RNAi lines and WT (**Figure S2**). Three independent RNAi transgenic lines (RNAi29, RNAi50 and RNAi60) with transcripts suppressed by over 80% (82.8% – 95.1%) in plantlets were selected for detailed further characterization. Since StInvInh1 possessed its inhibitory function *in vitro*, its cognate invertase activities were investigated in plantlets. Firstly, the acid invertase activities in the 10-day-old plantlets were visualized by a histochemical activity stain *in situ*. The NBT staining of the RNAi plantlets resulted in a darker blue than that of WT control, suggesting elevated acid invertase activities, while no color appearing in either RNAi plants or WT in the absence of substrate (sucrose) (**Figure 5A**). The invertase activity was then assayed *via* an enzyme assay *in vitro*. The results clearly indicated that only the CWI activities were increased significantly in the RNAi plantlets, while the activities of VI and CI showed little variation in comparison with WT control (**Figure 5B**). In addition, the expression levels of *CWI* genes (*StcwINV1* and *StcwINV2*) were not affected in the RNAi plantlets (**Figure 5C**). These results suggest that the CWI activity may be mostly regulated by StInvInh1 at post-translational level *in vivo*. Taken together, the results showed that the silencing of *StInvInh1* expression resulted in significant elevations of CWI activities in the RNAi plantlets, suggesting that StInvInh1 is a physiological target of CWI.

### Silencing *StInvInh1* expression enlarges size of micro-tuber *in vitro*

**Figure 6:**
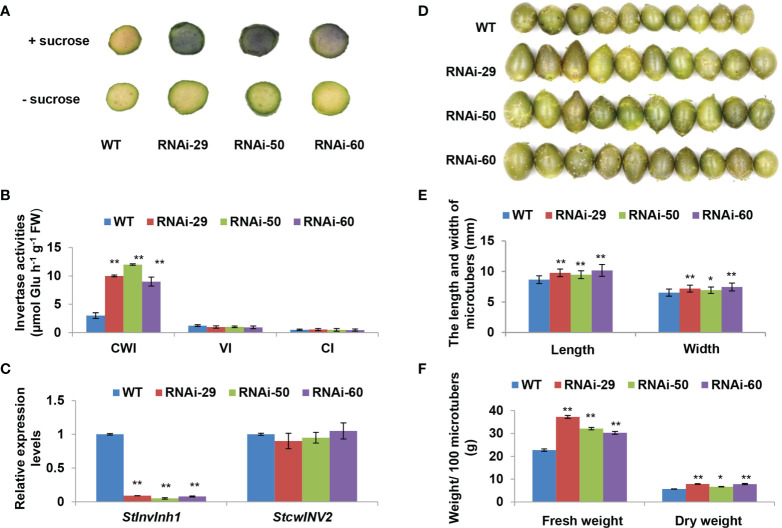
Performances of microtuber size and weight in RNAi lines. **(A)** Histochemical staining of NBT indicating the increased acid invertase activities of two-week-old microtubers in the RNAi lines. **(B)** The invertase activity determined by enzyme assay *in vitro* indicating the significantly increased the CWI activity in the RNAi micro-tubers without impacting the activities of VI and NI. **(C)** RT-qPCR analysis revealed that *StInvInh1* was suppressed in the RNAi micro-tubers without impact on mRNA levels of *StcwINV2*. **(D)** Performance of micro-tuber size in RNAi lines (10 four-week-old microtubers are shown in each line). **(E)** The length and width of micro-tubers in RNAi lines. **(F)** The fresh and dry weight of microtubers in RNAi lines. The relative expression levels of *StInvInh1* and *StcwINV2* are presented in relation to the expression levels of *ef1α* (AB061263) transcripts (100). The relative expression level of each gene and each enzyme activity in transgenic lines was compared with that in wild-type control E3. Each value was the mean ± SD of three biological replicates. Significant differences in values (^**^P < 0.01, ^*^P < 0.05) by Student’s t-test.

The characteristics of microtuberization were further investigated *in vitro*, and no significant difference was observed in either the percentage of microtuber formation by plantlets, the number of microtuber per plantlet, or the time of microtuber formation between WT and RNAi lines (data not shown). As excepted, significant increases in CWI activities were also observed in microtubers of the RNAi lines (**Figures 6A–C**). Interestingly, RNAi lines produced larger microtubers than the WT control (**Figure 6D**). The length and width of microtubers in RNAi lines were 9.37% –19.49% and 6.15% – 14.26% higher than that in WT control, respectively (**Figure 6E**). The length/width ratio in microtuber is similar between WT and RNAi lines. Compared with WT, the fresh weight of microtubers in RNAi lines increased by 33.35% – 64.15% with an evident increase in microtubers size. In addition, the dry weight of microtubers in RNAi lines also increased by 17.66% – 39.79%. However, the dry matter contents in two RNAi lines decreased significantly (**Table 1**). These findings demonstrate that the proportion of water content increased is higher than that of dry matter in microtuber production of RNAi lines. Furthermore, a significant increase in contents of sucrose, glucose and starch in the RNAi lines, while a little variation of fructose in comparison with the WT control (**Table 1**). These findings suggest that the elevated CWI activities are more closely associated with increases in size and dry matter production of microtubers.

**Table 1:** The contents of dry matter, sugar and starch in microtubers of RNAi lines.

Lines	Dry matter content (%)	Sucrose content(mg/g DW)	Glucose content(mg/g DW)	Fructose content(mg/g DW)	Starch content (%)
WT	24.91 ± 0.98	5.37 ± 0.07	2.37 ± 0.14	1.72 ± 0.50	30.28 ± 1.13
RNAi-29	21.21 ± 0.83^*^	7.43 ± 0.22^**^	3.74 ± 0.60^**^	1.78 ± 0.54	36.84 ± 1.18^**^
RNAi-50	20.71 ± 1.27^*^	8.77 ± 0.38^**^	3.16 ± 0.21^**^	1.75 ± 0.07	33.54 ± 1.05^*^
RNAi-60	25.85 ± 0.78	8.06 ± 0.13^**^	3.01 ± 0.72^**^	1.67 ± 0.15	38.81 ± 1.09^**^

Data represent mean ± SD of at least three biological replicates. Asterisks indicate significant differences in comparison with the WT as determined by Student’s t-test: **P < 0.01, *P < 0.05.

## Discussion

CWI-mediated sucrose metabolism and signaling is central to plant development (Ruan, 2014; Ruan, 2022). Apart from the transcriptional regulatory mechanism of invertase activities, emerging evidence also indicates that the subtle control of enzyme activities depends on the post-translational regulatory mechanism through interaction with their inhibitors (INHs) (Rausch and Geiner, 2004). Although the INH was initially discovered in potato as early as in the 1960s (Schwimmer et al., 1961; Pressey and Shaw, 1966), the corresponding cDNAs from *Nicotiana tabacum* was cloned until late 1990s (Greiner et al., 1998; Greiner et al., 1999). Sequence analyses *in silico* suggested that the INH family is moderately conserved within different plant species (Rausch and Greiner, 2004). Both INHs and PMEIs (pectin methylesterase inhibitors) belong to the same superfamily named PMEI-related protein based on their similar protein structure, enabling it’s difficult to distinguish them from sequence comparisons (Hothorn et al., 2004).

In our previous study, four cDNAs encoding putative INHs were isolated in potato. Among them, both StInvInh2A and StInvInh2B were identified as INHs of VI and play roles in regulating potato CIS by capping VI activity (Liu et al., 2010; Liu et al., 2013; Lin et al., 2015). Based on sequence phylogenetic analyses and subcellular localization, the other two putative INHs, StInvInh1 and StInvInh3, were expected as targets of CWIs (Liu et al., 2010). In combination with RNA-seq data, the RT-qPCR analyses of spatiotemporal expression of *StInvInh1* and *StInvInh3* revealed that *StInvInh1* showed an overall higher expression in all tested organs (**Figure 1**), indicating that *StInvInh1* may be an important putative *INH* gene related to biological processes in potato growth and development. Interestedly, interaction between StInvInh1 and VI/CWIs was identified in potato using modelling approaches (Datir and Ghosh, 2020). The targets of INHs need to be clarified through both *in vitro* and *in vivo* approaches (Liu et al., 2010; Liu et al., 2013; Coculo and Lionetti, 2022). In this study, enhanced evidences demonstrated that StInvInh1 functions as an INH of CWI in potato. Firstly, the activity of CWI protein from potato was inhibited by the recombinant StInvInh1 protein *in vitro* (**Figure 4B**). Secondly, the interaction of StInvInh1 and StcwINV2 was confirmed by the BiFC in BY-2 cells (**Figure 4C**), indicating that StInvInh1 targets CWI *in situ*. Finally, silencing the expression of *StInvInh1* elevated the CWI activity without having impact on expression levels of *CWIs*, suggesting that a high proportion of CWI activity is under post-translational control of StInvInh1 in potato. In addition, altered *StInvInh1* expression did not affect activities of VI and CI (**Figure 5**), indicating a high specificity of StInvInh1 in capping CWI activity. Collectively, these data strongly indicated that StInvInh1 functions as an INH of CWI in potato.

It is reported that INHs of CWI were shown to be ABA-responsive genes and predominantly expressed in flowers and seeds (Jin et al., 2009; Yang et al., 2020). The seed weight and production were improved by silencing or knock-out of *INHs* in tomato, *Arabidopsis*, and soybean (Jin et al., 2009; Su et al., 2016; Tang et al., 2017). Similarly, *StInvInh1* was also found to have the highest expression in flowers as well as in response to ABA (**Figures 1**, **2**), suggesting its potential conserved role in seed development. In addition, the expression of *StInvInh1* appeared to decrease progressively with tuber development (**Figures 1B**, **3C, D**), providing evidence of its potential role in tuber development. The critical role of CWI in sinks has been demonstrated through mutational and transgenic analyses. An endosperm-specific *CWI* gene mutant in maize resulted in miniature seeds (Cheng et al., 1996). The phenotype is probably caused by the suppression of auxin biosynthesis (LeClere et al., 2010) and reduced mitotic activity and cell size in the endosperm (Vilhar et al., 2002). A similar role for CWI in seed development was found in rice (Wang et al., 2008) and tomato (Zanor et al., 2009). However, it remains a debate whether and how CWI activity plays a role in tuber formation and development, a prerequisite for tuber production. Initially, the constitutive overexpression of the yeast invertase gene in apoplast caused the plants to appear to be under stress and yield penalty (Heineke et al., 1992; Büssis et al., 1997). Subsequently, the tuber-specific overexpression of yeast invertase gene in apoplast resulted in increased tuber size and total yield due to an increase in water content (Sonnewald et al., 1997; Hajirezaei et al., 2000; Ferreira and Sonnewald, 2012). Although these approaches to increasing CWI activities have been studied for the formation and development of tubers, the improvement of dry matter production in potato tubers seemed a failure. CWIs were co-evolved with vascular plants with the gene family expansion in seed plants from gymnosperm to angiosperm (Wan et al., 2018). CWI was reported to be encoded by multiple genes which have distinct but partially overlapping expression patterns in potato (Liu et al., 2011), suggesting a unique function for individual gene. These reports suggest that CWI activity need to be tightly regulated to balance development and stress adaptation for tuber production improvement in potato. Here, transgenic potato plants were generated by RNAi-mediated silencing of *StInvInh1* in order to investigate its effects on tuber formation and development. The microtuberization characteristics of RNAi lines were investigated *in vitro*. Interestingly, the specific suppression of *StInvInh1* expression significantly improved the size, fresh weight, and dry matter production of microtubers with remarkable elevated CWI activities (**Figure 6**). The results were consistent with the previous reports that the seed weight and production were improved by silencing or knock-out of *INHs* in tomato, *Arabidopsis*, and soybean (Jin et al., 2009; Su et al., 2016; Tang et al., 2017). The improvement of dry matter production of microtubers could result from the elevation of CWI activity during the early stages of tuber formation, because the class I patatin B33 promoter used to design tuber-specific constructs appears to be inactive in stolon and during the early stages of tuber formation (Tauberger et al., 1999). CWI could contribute to sink development by facilitating phloem unloading of sucrose and converting it to glucose and fructose as major nutrients and energy sources. Compared with WT, the sucrose and glucose contents were significantly increased in microtubers of RNAi lines with elevation of CWI activity (**Table 1**), probably promoting phloem unloading of sucrose. Moreover, CWI-mediated signaling can modulate the expression of sugar transporter and regulatory genes (Ru et al., 2017; Liao et al., 2020). Similarly, functional loss of *SlInvInh1* in tomato by genome editing increased sugar content of fruit (Kawaguchi et al., 2021). In this study, no obvious difference in fructose content was observed between WT and RNAi lines (**Table 1**). One possible explanation is that the utilization of fructose also was activated in RNAi lines, which resulting in a balance between fructose production and utilization. These findings, together with evidence of glucose positively regulating cell division (Weber et al., 2005) indicate a role of CWI activity in early tuber development, which could partially explain a bigger microtuber size phenotype under elevation of CWI activity in microtubers. Tuberization in potato involves a switch from CWI-mediated apoplastic to Susy-mediated symplastic phloem unloading (Viola et al., 2001). However, no significant difference was observed in either the percentage of microtuber formation by plantlets, the number of microtuber per plantlet, or the time of microtuber formation between WT and RNAi lines (data not shown). It cannot be ruled out that the possibility of differences from tuberization conditions between *in vitro* and *in vivo.* The potential roles of *StInvInh1* in potato growth and development *in vivo* will be the subject of future investigations.

## Conclusion

Emerging evidence has indicated that the CWIs play fundamental roles in plant reproductive development as well as the regulation of sucrose metabolism and homeostasis through fine-tuning the CWI activities. In this study, enhancive evidences demonstrate that StInvInh1 functions as an INH of CWI in potato, which results in an impact on microtuber development *in vitro*. In the future, we will confirm the roles of *StInvInh1* in potato growth and development *in vivo* and decipher the molecular evidence. Our results provide developmental evidence that *StInvInh1* plays a vital role in microtuber development in potato, which may promise great potential to improve tuber performance through manipulation of CWI activity in potato.

## Data availability statement

The original contributions presented in the study are included in the article/**Supplementary Material**. Further inquiries can be directed to the corresponding author.

## Author contributions

XL conceived and designed the experiments. CL performed most of the experiments. DX and SH performed the transformation and tuberization of potato. QC and SL helped with the promoter activity analysis. WS, LS, HS, and ZL helped with the vectors construction and biochemical analysis. XL and CL wrote the manuscript. RJ, DL, and JW were involved in data analysis and proofreading. All the authors discussed the results and collectively edited the manuscript. All authors contributed to the article and approved the submitted version.

## Funding

This work was supported by the National Key Research and Development Program (NKRDP) (2018YFE0127900), the Natural Science Foundation of China (NSFC) (31571728), the Undergraduate Innovation and Entrepreneurship Training Program in SWU (UIETP) (202210635030), and the Science and Technology Partnership Program (STPP) (KY201904016).

## Acknowledgments

The authors would like to thank NKRDP, NSFC, UIETP and STPP for funding this research. We are also grateful to Prof. Zhihua Liao from Southwest University for donating the pGreenII 0800-LUC vector.

## Conflict of interest

The authors declare that the research was conducted in the absence of any commercial or financial relationships that could be construed as a potential conflict of interest.

## Publisher’s note

All claims expressed in this article are solely those of the authors and do not necessarily represent those of their affiliated organizations, or those of the publisher, the editors and the reviewers. Any product that may be evaluated in this article, or claim that may be made by its manufacturer, is not guaranteed or endorsed by the publisher.
