# Supplementary material for: Functional characterization of a cell wall invertase inhibitor StInvInh1 revealed its involvement in potato microtuber size in vitro
Source: Front Plant Sci. 2022 Oct 3;13:1015815. doi: 10.3389/fpls.2022.1015815 (PMC9574400; doi:10.3389/fpls.2022.1015815)
Supplement: Supplementary file 1 [file DataSheet_1.docx]

**Supplementary Materials**

Table S1 The sequences of the PCR primers for RT-qPCR

| Gene | Primer sequence (5'-3') | References |  |
| --- | --- | --- | --- |
| *ef1α* | ATTGGAAACGGATATGCTCCA | Nicot et al., 2005 |  |
| *ef1α* | TCCTTACCTGAACGCCTGTCA |  |  |
| *StInvInh1* | GACAAAAGAAGTGAAACAGCAGGA | Liu et al., 2010 |  |
| *StInvInh1* | GAGGATTAGAATGCCTAAGCTTTGA |  |  |
| *StInvInh3* | TGGCCGGTTTGAAATATGTTCTT | Liu et al., 2010 |  |
| *StInvInh3* | TGTAGCTGGTCTTACAGATGTC |  |  |
| *StcwINV1* | TGTTATCGGGCTTGATCCATT | Liu et al., 2011 |  |
| *StcwINV1* | ATCCCACTTGAACCCGCTATC |  |  |
| *StcwINV2* | TGTTGCCTGGCAAGATTGTG | Liu et al., 2011 |  |
| *StcwINV2* | AAGTTTGAACAAGGCCGAACA |  |  |
| *GUS* | AGGTGCACGGGAATGTTTCG | Liu et al., 2017 |  |
| *GUS* | CAATAAATTTCTTACAATAGCTCTACC |  | |


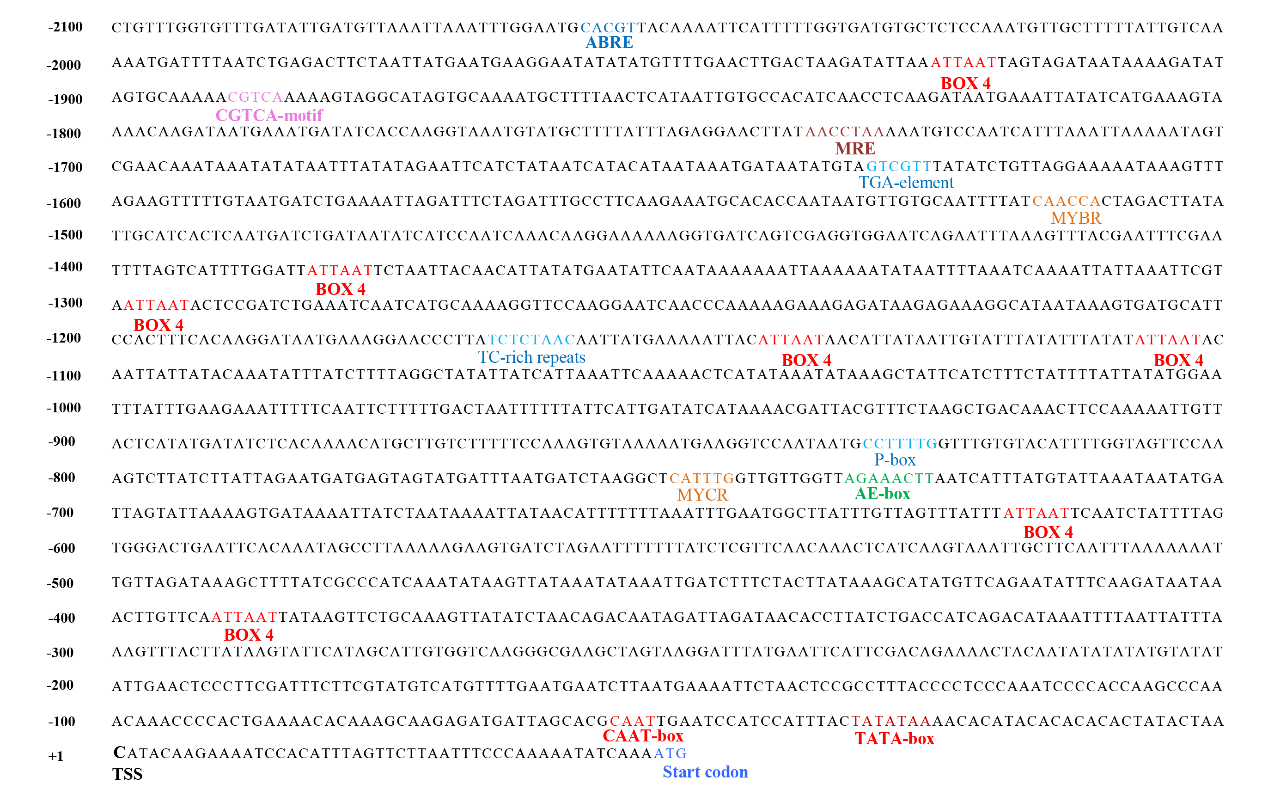


**Figure S1 The 5 -flanking sequence of *StInvInh1***

The start codon, transcription start site (TSS), TATA-box, CAAT-box, P-box, ABA-repressive element (ABRE), MeJA-responsive element (CGTCA-motif), and so on are colored. Numbers indicate the position relative to the TSS.


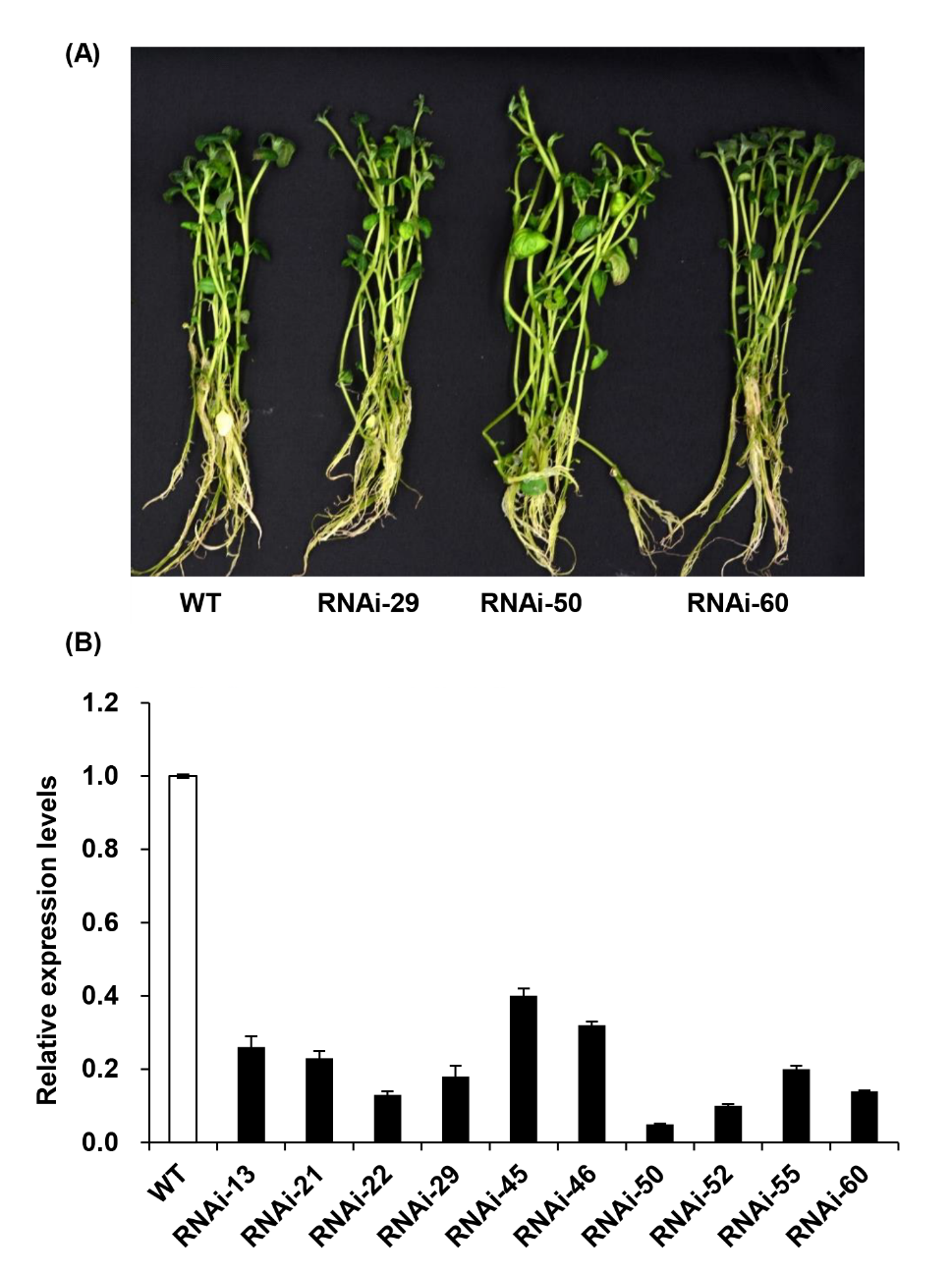


**Figure S2 Representative transgenic lines**

A: Images of four-week-old plantlets in RNAi lines; B: The relative expression levels of *StInvInh1* in four-week-old plantlets of RNAi lines. The relative expression level of *StInvInh1* in RNAi lines was compared with that in wild-type control E3.
